# Functional characterization of HbRAR1 in *Hevea brasiliensis* reveals its role in the HSP90–SGT1–RAR1 complex during hypersensitive response

**DOI:** 10.3389/fpls.2026.1751305

**Published:** 2026-02-03

**Authors:** Qifeng Liu, Jiali Wang, Fei Yu, Yiying Lu, Yu Zhang, Meng Wang, Xiaoyu Liang

**Affiliations:** 1 State Key Laboratory of Tropical Crop Breeding, Sanya Institute of Breeding and Multiplication, School of Tropical Agriculture and Forestry, Hainan University, Sanya, China; 2 Huizhou Customs, Huizhou, Guangdong, China

**Keywords:** HbCNL4, HbHSP90.1, HbRAR1, HbSGT1b, *Hevea brasiliensis*, HR

## Abstract

Powdery mildew is one of the most destructive foliar diseases of rubber tree (*Hevea brasiliensis*). To clarify the role of the conserved HSP90**–**SGT1**–**RAR1 molecular complex in rubber tree immunity, we identified and characterized two RAR1 homologs, HbRAR1–1 and HbRAR1-2. Both genes were significantly induced upon powdery mildew (*Erysiphe quercicola*) infection and were localized to the cytoplasm and nucleus. Yeast two-hybrid and bimolecular fluorescence complementation assays demonstrated that the HbRAR1 proteins interact with HbHSP90.1 and HbSGT1b via their two CHORD domains. Specifically, HbRAR1 binds the HATPase domain of HbHSP90.1 and the CS domain of HbSGT1b, forming a canonical HSP90**–**SGT1**–**RAR1 ternary complex. Additionally, Y2H, and BiFC assays confirmed that the core components of this complex, HbHSP90.1 and HbSGT1b, associate with the NLR immune receptor HbCNL4. Transient overexpression and electrolyte leakage assays indicate that co-expression of the HSP90**–**SGT1**–**RAR1 ternary complex with HbCNL4 enhances the hypersensitive response induced by HbCNL4. This study provides the first evidence in rubber tree that the HSP90**–**SGT1**–**RAR1 complex is structurally and functionally conserved and may play a central role in modulating NLR-triggered immune signaling. These findings offer key mechanistic insights into powdery mildew resistance and supply novel genetic resources for the cultivation of disease-resistant rubber tree cultivars.

## Introduction

Powdery mildew, caused by *Erysiphe quercicola*, is one of the most destructive foliar diseases affecting rubber tree (*Hevea brasiliensis*) plantations in China. Severe infections can result in yield losses of up to 45%, representing a major challenge to latex production, particularly in key rubber-producing regions (Li et al., 2023; Cao et al., 2024). The development of disease-resistant cultivars offers a sustainable solution for controlling this disease, making the identifying of resistance genes and understanding their underlying molecular mechanisms essential.

In plants, Nucleotide-binding leucine-rich repeat (NLR) proteins play a pivotal role as primary immune receptors, recognizing pathogen effectors and triggering effector-triggered immunity (ETI) (Duggan et al., 2021). This often leads to a hypersensitive response (HR), a form of programmed cell death that limits pathogen spread and enhances resistance (Huang et al., 2023; Sun et al., 2024).

The RAR1 (required for MLA12 resistance) gene was first identified in barley during studies of MLA12-mediated resistance to powdery mildew (Shen et al., 2003; Bieri et al., 2004). The RAR1 protein contains two highly conserved zinc-binding domains, termed CHORD-I and CHORD-II (cysteine- and histidine-rich domains), which are separated by a 20-amino acid linker harboring three cysteine residues and one histidine (CCCH motif) (Zhang et al., 2010; Hong et al., 2013). Each CHORD domain coordinates one zinc ion, and these zinc ions are essential for proper protein folding and structural stability (Hong et al., 2013). Accumulating evidence indicates that RAR1 is a critical component of immune signaling in both monocotyledonous and dicotyledonous plants. It functions as a key regulator in powdery mildew resistance pathways and is required for the activity of multiple resistance (R) genes against diverse pathogens (Shen et al., 2003). In barley and rice, *rar1* mutants fail to accumulate reactive oxygen species and do not exhibit an HR upon pathogen challenge, resulting in heightened susceptibility to *Blumeria graminis* and *Magnaporthe grisea* (Shen et al., 2003; Jarosch et al., 2005).

Accumulating studies have established that the molecular chaperone complex composed of HSP90, SGT1, and RAR1 serves as a central hub in regulating plant innate immunity (Seo et al., 2008; Ito et al., 2015; Zhang et al., 2015; Katsuyama et al., 2021; Yuan et al., 2021). This complex plays a critical role in plant defense by stabilizing NLR immune receptors and modulating their activation during pathogen invasion. RAR1 interacts directly with both HSP90 and SGT1. This interaction is essential for maintaining the stability of certain resistance proteins (e.g., RPS2 in Arabidopsis, Rx in potato), thereby contributing significantly to immune signaling (Takahashi et al., 2003; Boter et al., 2007). In wheat, for example, Wang et al. demonstrated that the interaction between TaRAR1, TaSGT1, and TaHSP90 enhances *R* gene-mediated resistance to stripe rust (Wang et al., 2015). Similarly, in barley, resistance to powdery mildew triggered by the MLA6 NLR receptor is dependent on functional RAR1 and SGT1 (Shen et al., 2003). In *Solanum lycopersicum*, the NLR protein Sw-5b confers resistance to tomato spotted wilt virus (TSWV) in an SGT1-dependent manner (Chen et al., 2021). These findings collectively highlight that while the involvement of HSP90, SGT1, and RAR1 in immune responses is broadly conserved, the modes of their interaction and their specific roles in immunity can vary depending on the NLR protein and the context of the pathogen.

In rubber tree, eight *HSP90* genes have been identified, with HbHSP90.1 functioning as a molecular chaperone for SGT1 and playing a key role in disease resistance (Liu et al., 2022). HbSGT1b interacts with HbHSP90.1 in the nucleus, and HbHSP90.1 also associates with the NLR protein HbCNL2, further supporting the involvement of this chaperone complex in immune signaling (Wang et al., 2023). Additionally, the NLR protein HbCNL4 is specifically induced in rubber tree epidermal cells following pathogen invasion, and it triggers HR in multiple plant species. This suggests HbCNL4 likely functions downstream of the HSP90**–**SGT1**–**RAR1 complex (Liang et al., 2023; Zhang et al., 2025). However, the mechanism by which the HSP90**–**SGT1**–**RAR1 complex interacts with NLR proteins in woody plants remains poorly understood, and the specific role of RAR1 in activating CNL-type NLRs in the rubber tree is unknown. Elucidating these interactions will deepen our understanding of immune receptor regulation in perennial species and provide molecular targets for breeding disease-resistant rubber trees.

## Methods

### Plant materials

Plant materials of the rubber tree cultivar ‘Reyan 73397’, a high-yielding cultivar widely promoted in China, were obtained from the germplasm nursery of the Rubber Research Institute, Chinese Academy of Tropical Agricultural Sciences. Roots, stems, branches, leaves, latex, and flowers from healthy tapping trees were collected for tissue-specific expression analysis. Grafted ‘Reyan 73397’ seedlings were used for powdery mildew inoculation to assess gene expression under pathogen challenge. Fresh spore suspension of *E. quercuola* HO-1 strain was used to infect the leaves of rubber tree seedlings at the bronze stage, followed by placing the treated rubber tree seedlings in a stable environment with a temperature of 22-24°C, 60%-80% RH, and a light-dark cycle of 14 h: 10 h. Untreated plants served as controls. Each experiment was conducted three times, and all collected samples were immediately frozen in liquid nitrogen and stored at -80°C for further analysis. *Nicotiana benthamiana* seeds used in this study were maintained in our laboratory.

### Characterization and analysis of the *HbRAR1*

Based on the published RAR1 protein sequences of *Arabidopsis* and *Manihot esculenta* available in the NCBI database, two homologous HbRAR1 sequences were identified from the rubber tree. The conserved domains of the two HbRAR1 proteins were analyzed using the NCBI Conserved Domain Database (Batch CD-Search; https://www.ncbi.nlm.nih.gov/Structure/bwrpsb/bwrpsb.cgi, October 26, 2024). Physicochemical characteristics, including molecular weight (MW) and isoelectric point (pI), were predicted using the ExPASy ProtParam tool (https://web.expasy.org/protparam/, October 22, 2025). To examine the evolutionary relationships, a phylogenetic tree was constructed using the maximum likelihood method in MEGA-X software with 1,000 bootstrap replicates.

### Analysis of HbRAR1 expression patterns

Total RNA was extracted from powdery mildew-infected rubber tree leaves at different time and from various tissues of *H. brasiliensis* using a Plant Total RNA Extraction Kit (Takara, Tokyo, Japan). First-strand cDNA was synthesized from the extracted RNA using a reverse transcription kit (Thermo Fisher, Beijing, China). HbActin served as the internal reference gene for expression normalization.

### Plasmid construction

The pBin–Myc vector was used to construct HbCNL4, HbHSP90.1, HbSGT1b, HbRAR1-1, and HbRAR1–2 for transient overexpression. For subcellular localization, GFP-tagged HbRAR1–1 and HbRAR1–2 were cloned into the pCAMBIA1300 vector. Coding sequences were amplified from rubber tree cDNA or genomic DNA using gene-specific primers (**Supplementary Table S1**) and inserted into the corresponding vectors via homologous recombination. For yeast two-hybrid (Y2H) assays, full-length and truncated forms of HbCNL4, HbHSP90.1, HbSGT1b, HbRAR1-1, and HbRAR1–2 were cloned into pGBKT7 and pGADT7 vectors. The same fragments were subcloned into pSPYNE and pSPYCE vectors for bimolecular fluorescence complementation (BiFC) assays.

### Subcellular localization of HbRAR1

Fully expanded leaves from healthy 3–4 weeks-old tobacco plants were used for transient expression assays. Agrobacterium tumefaciens strains carrying GFP-tagged HbRAR1–1 or HbRAR1–2 constructs were infiltrated into the abaxial side of the leaves. Following 48 hours of infiltration, the leaves were harvested and observed for green and red fluorescence signals under excitation light at a wavelength of 488 nm and 543 nm using Zeiss LSM 880 confocal microscope (Zeiss LSM 880, Jena, Germany).

### Yeast two-hybrid assay

For yeast two-hybrid (Y2H) assays, recombinant pGBKT7 and pGADT7 plasmids carrying the respective genes were co-transformed into yeast cells. transformed yeast cells were first selected on SD/-Trp/-Leu medium at 28°C for 3 days. Single colonies were then suspended in sterile ddH_2_O and spotted onto SD/-Trp/-Leu/-His/-Ade plates, followed by incubation at 30°C for 3–5 days.

### Bimolecular fluorescence complementation assay

Agrobacterium tumefaciens GV3101 strains carrying the respective constructs were resuspended in infiltration buffer (10 mM MgCl_2_, 10 mM MES, 200 μM acetosyringone, pH 5.6) and adjusted to an OD_600_ of 1.0. Equal volumes of Agrobacterium cultures containing paired gene constructs were mixed (1:1) and infiltrated into tobacco leaves. YFP signals in epidermal cells on the abaxial side were examined 1–3 days post-infiltration using a Zeiss LSM 880 confocal microscope.

### Hypersensitive response assay

Agrobacterium tumefaciens GV3101 strains carrying the recombinant constructs were resuspended in infiltration buffer and adjusted to an OD_600_ of 0.5. The suspensions were infiltrated into fully expanded tobacco leaves, and HR symptoms were recorded 1–3 days post-infiltration. Ion leakage was quantified according to the method described by Nie et al. (2019). Six leaf discs were immersed in 5 mL of deionized water (ddH_2_O) and incubated for 5 hours. Conductivity (Value A) was measured using a conductivity meter (FE32 FiveEasy; Mettler-Toledo). The samples were then boiled for 20 minutes, cooled to room temperature, and remeasured (Value B). Ion leakage was calculated as (A/B) × 100%. The experiment was performed in triplicate. Ion leakage data were analyzed using a two-tailed Student’s t-test. All statistical analyses were conducted using OriginPro 2018 (OriginLab).

## Results

### Characterization and analysis of the *HbRAR1*

Two RAR1 genes, designated HbRAR1–1 and HbRAR1-2, were identified from the rubber tree genome database. The full-length sequence of HbRAR1–1 is 1046 bp, containing a 657 bp open reading frame (ORF) that encodes a 218-amino-acid protein. HbRAR1–2 is 1091 bp in length, with a 651 bp ORF corresponding to 216 amino acids. Physicochemical characterization using the ExPASy-ProtParam tool revealed that the theoretical molecular mass of HbRAR1–1 is 24.21 kDa with an isoelectric point (pI) of 8.32 and an average hydropathicity of –0.794. HbRAR1–2 has a predicted molecular mass of 23.86 kDa, a pI of 8.33, and an average hydropathicity of –0.792. The instability indices for HbRAR1–1 and HbRAR1–2 were 51.07 and 51.49, respectively, indicating both proteins are classified as unstable.

**Figure 1:**
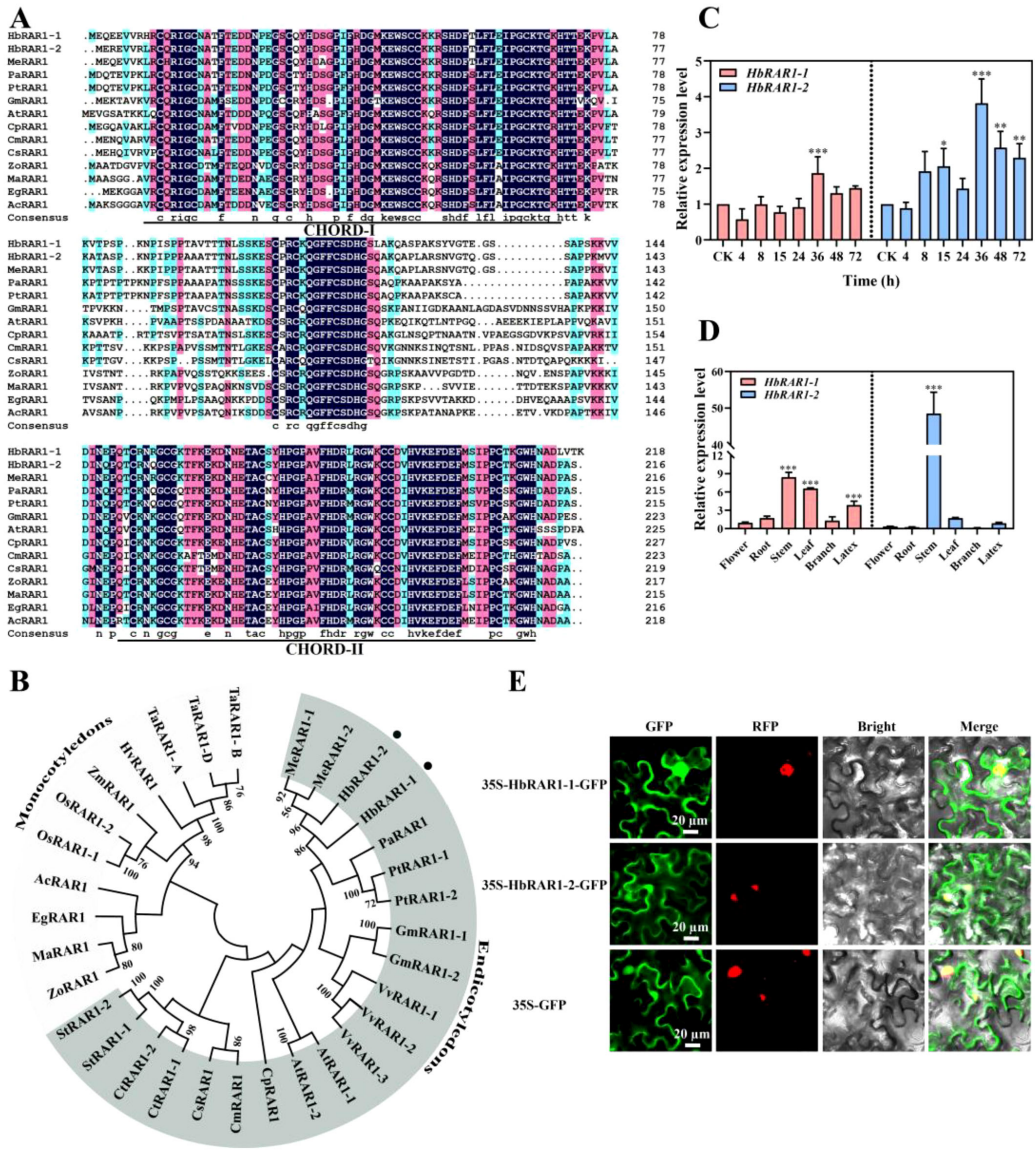
Identification and characterization of *HbRAR1* genes. **(A)** Multiple sequence alignment of HbRAR1. **(B)** Phylogenetic tree of HbRAR1 proteins. Colored boxes indicate monocot and dicot species. Black circles highlight the two HbRAR1 proteins. **(C)** Expression profiles of *HbRAR1* genes at different time following powdery mildew infection. **(D)** Tissue-specific expression patterns of *HbRAR1* genes in rubber tree. Error bars represent ± SD of the values from three biological repeats. Statistical significance was determined using Student’s t-test. *P < 0.05, **P < 0.01, ***P < 0.001. **(E)** Subcellular localization of HbRAR1 in the plasma membrane and nucleus. 35S-GFP serves as the empty vector control. Bright, Images show the bright field; GFP, green fluorescence; RFP, red fluorescence; Merge, merged signals; Scale bar, 20 μm.

Multiple sequence alignment of HbRAR1–1 and HbRAR1–2 with RAR1 proteins from other plant species showed that all RAR1 orthologs possess two conserved CHORD domains, designated CHORD-I and CHORD-II (**Figure 1A**). Among the species analyzed, rubber tree RAR1 proteins showed the highest amino acid identity with cassava RAR1 (MeRAR1). HbRAR1–1 and HbRAR1–2 exhibited 86.70% and 93.52% identity with MeRAR1, whereas sequence identity between HbRAR1–1 and HbRAR1–2 was 88.99%. Phylogenetic analysis groups the HbRAR1 proteins closely with cassava RAR1 in the same clade, distinct from RAR1 of monocots and other dicots (**Figure 1B**).

### Expression patterns of *HbRAR1*

qRT-PCR analysis reveals that both *HbRAR1–1* and *HbRAR1–2* were transcriptionally induced after *E. quercicola* inoculation, with expression progressively increasing and peaking at 36 hpi. At this time, *HbRAR1–1* expression was approximately 1.9-fold higher than pre-inoculation levels, while *HbRAR1–2* expression was approximately 3.8-fold higher, indicating that the *HbRAR1* genes are primarily involved in the mid-to-late stages of the defense response (**Figure 1C**).

Tissue-specific expression analysis showed that the two genes exhibited similar expression profiles across organs (**Figure 1D**). Both were most highly expressed in stems, followed by leaves, latex, branches, and flowers, suggesting that *HbRAR1* functions primarily in stems and other metabolically active tissues of rubber tree.

### Subcellular localization of *HbRAR1* proteins

Subcellular localization analysis (**Figure 1E**) showed that both HbRAR1–1 and HbRAR1–2 fluoresced in the nucleus and cytoplasm, indicating these proteins are present in both the nucleus and cytoplasm, and thus may function in both cellular compartments.

### HbRAR1 interacts with HbHSP90.1 and HbSGT1b via its CHORD domains

**Figure 2:**
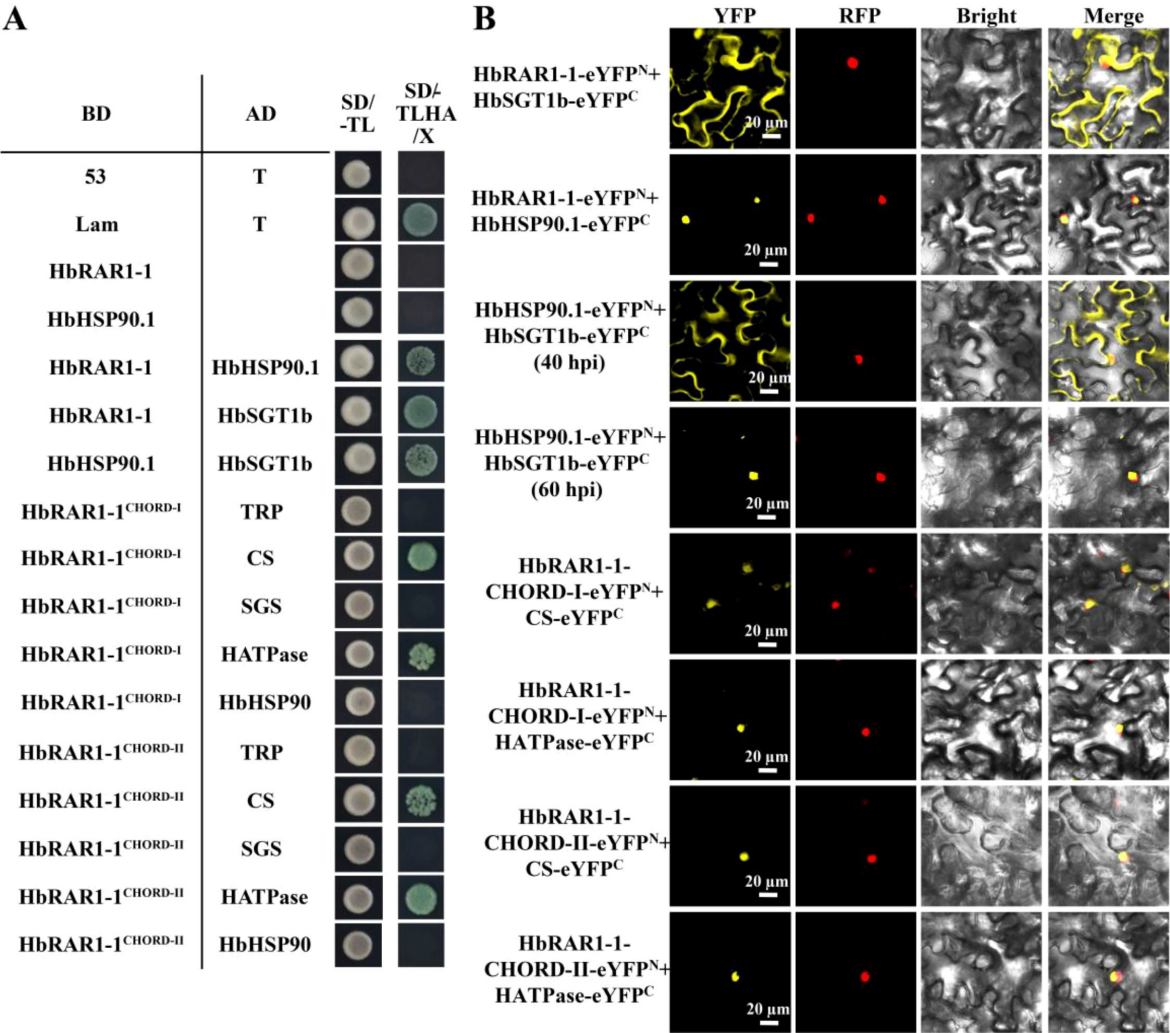
Interaction of HbRAR1–1 with HbHSP90.1 and HbSGT1b. **(A)** Y2H assays show that HbRAR1–1 interacts with HbHSP90.1 and HbSGT1b through the CHORD-I and CHORD-II domains of HbRAR1-1, the HATPase domain of HbHSP90.1, and the CS domain of HbSGT1b. **(B)** BiFC assays further confirm these interactions, demonstrating that HbRAR1-1, HbHSP90.1, and HbSGT1b associate via the CHORD-I, CHORD-II, HATPase, and CS domains. Scale bar, 20 μm.

**Figure 3:**
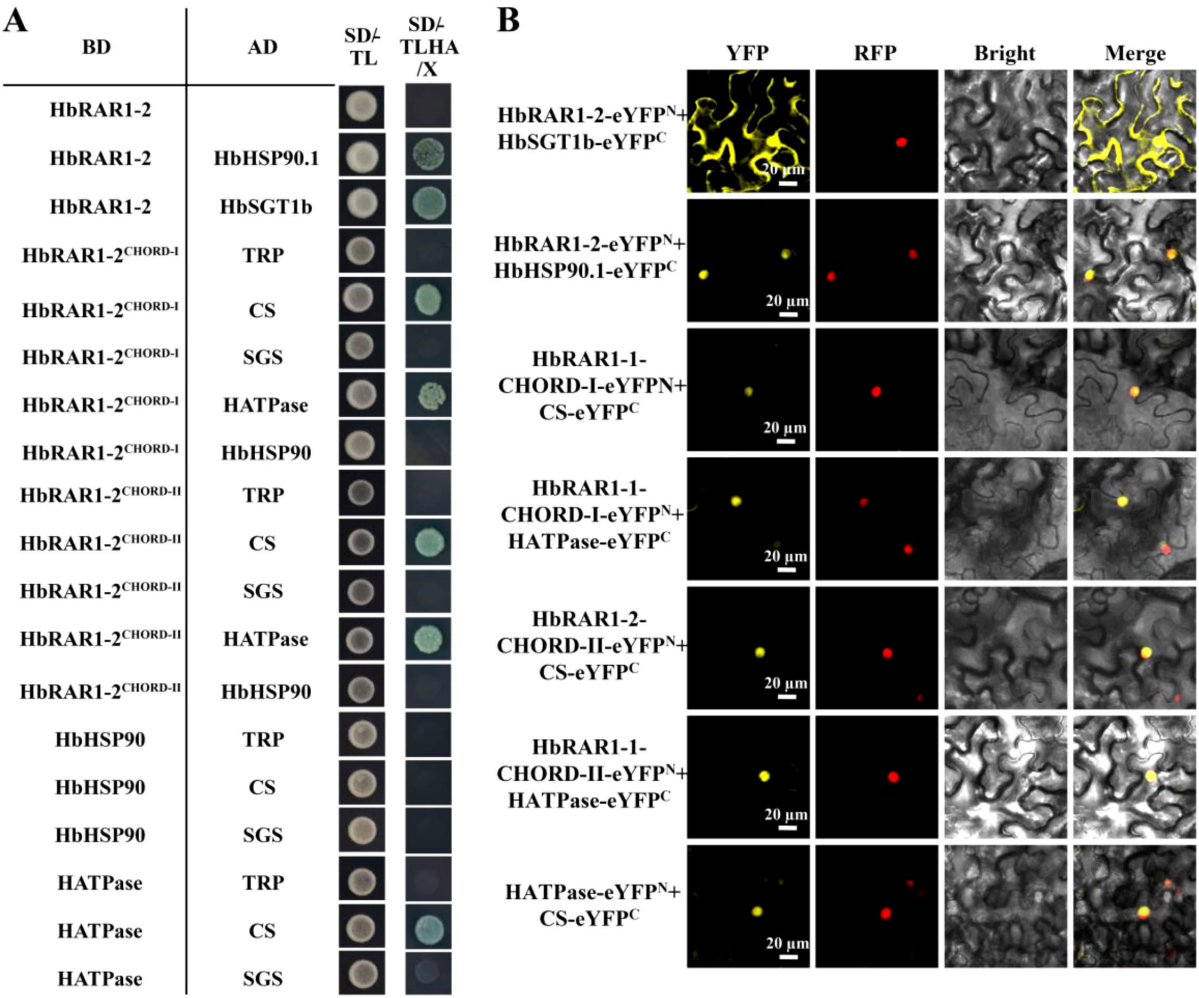
Interaction of HbRAR1–2 with HbHSP90.1 and HbSGT1b. **(A)** Y2H assays indicate that HbRAR1–2 interacts with HbHSP90.1 and HbSGT1b through the CHORD-I and CHORD-II domains of HbRAR1-2, the HATPase domain of HbHSP90.1, and the CS domain of HbSGT1b. **(B)** BiFC assays confirm that HbRAR1-2, HbHSP90.1, and HbSGT1b associate *via* the CHORD-I, CHORD-II, HATPase, and CS domains. Scale bar, 20 μm.

To investigate whether HbRAR1, HbHSP90.1, and HbSGT1b form a ternary complex, Y2H and BiFC assays were performed. The results showed that both HbRAR1–1 and HbRAR1–2 interact with HbHSP90.1 and HbSGT1b (**Figures 2A, B**; **Figures 3A, B**; **Supplementary Figures S1A, B**). To identify the interaction domains, truncated variants of the three proteins were constructed and analyzed. Both HbRAR1 proteins interacted with the HATPase domain of HbHSP90.1 and the CS domain of HbSGT1b *via* their two conserved CHORD domains. These data indicate that the CHORD domains, together with the HATPase domain and the CS domain, are required for assembly of the HbRAR1–HbHSP90.1–HbSGT1b ternary complex in the rubber tree.

### HbHSP90.1 and HbSGT1b interact with HbCNL4

**Figure 4:**
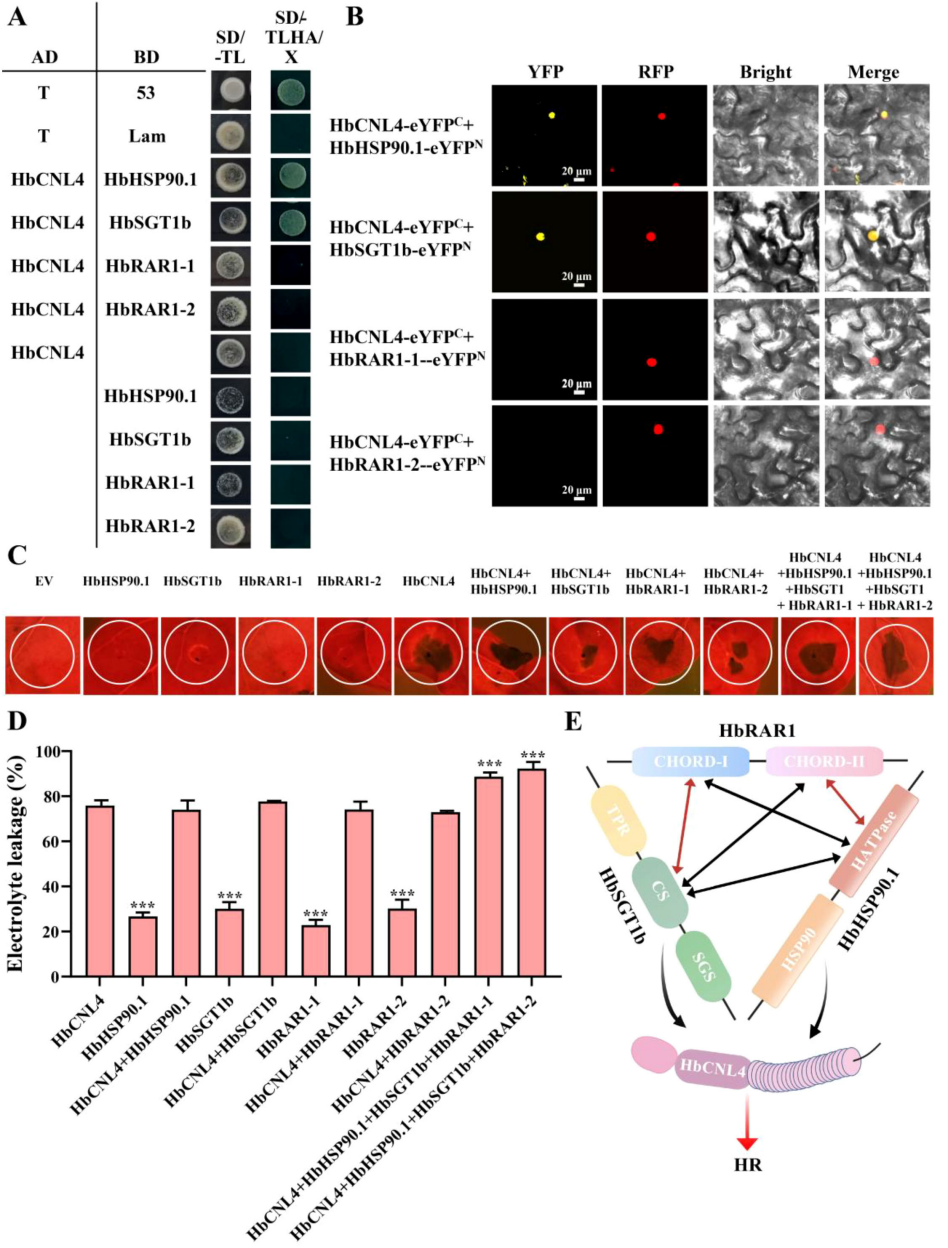
The HbRAR1–HbHSP90.1–HbSGT1b ternary complex enhances HbCNL4-mediated hypersensitive response (HR). **(A)** Y2H assays show that HbCNL4 interacts with HbHSP90.1 and HbSGT1b. **(B)** BiFC assays confirm the interactions between HbCNL4 and HbHSP90.1 or HbSGT1b. Scale bar, 20 μm. **(C)** Transient overexpression of HbCNL4 and the HbRAR1**–**HbHSP90.1**–**HbSGT1b complex in *N. benthamiana*. EV refers to the single injection of the pBin empty vector. **(D)** Electrolyte leakage assay evaluating HR intensity. Error bars represent ± SD of the values from six biological repeats. Statistical significance was determined using Student’s t-test. ***P < 0.001. **(E)** Proposed model of the HbRAR1**–**HbHSP90.1**–**HbSGT1b ternary complex promoting HbCNL4-triggered HR in rubber tree.

Single-cell transcriptome analysis showed that *HbCNL4* is strongly induced after *E. quercicola* infection and HbCNL4 induces HR in multiple plant species, suggesting its involvement in immune responses (Liang et al., 2023; Zhang et al., 2025). As the HSP90**–**SGT1**–**RAR1 complex is known to stabilize and regulate NLR proteins in other plants, we investigated whether a similar mechanism operates in rubber tree. Y2H and BiFC assays demonstrated that HbCNL4 interacts with both HbHSP90.1 and HbSGT1b (**Figures 4A, B**; **Supplementary Figure S1C**). This suggests that the HSP90.1**–**SGT1b**–**RAR1 complex may contribute to stabilizing and functionally regulating HbCNL4 in rubber tree.

### HSP90-SGT1-RAR1 ternary complex enhances HbCNL4-triggered HR

Previous experiments demonstrated that HbCNL4 interacts with components of the rubber tree HSP90**–**SGT1**–**RAR1 ternary complex, implying that this complex may play a positive role in HbCNL4-mediated immunity. To test this, we transiently co-expressed HbCNL4 with individual or combined complex components in tobacco. The individual injection of HbRAR1-1, HbRAR1-2, HbSGT1b, and HbHSP90.1 did not induce tobacco cell death. However, overexpression of HbCNL4 alone, or its co-expression with HbHSP90.1, HbSGT1b or HbRAR1 individually, all triggered a HR in tobacco leaves. Electrolyte leakage assays revealed that co-expression of HbCNL4 with HbHSP90.1, HbSGT1b, and HbRAR1 together resulted in a significantly stronger HR (**Figures 4C, D**). In contrast, no such enhancement was observed when HbCNL4 was co-expressed separately with any single one of these three genes. This suggests that all three components of the complex may be required for full HbCNL4-mediated defense signaling.

## Discussion

RAR1 is a core component of plant immune signaling and functions in multiple NLR-mediated pathways. However, its role in rubber tree has not been fully explored. In this study, we identified two homologs of the *RAR1* gene, *HbRAR1–1* and *HbRAR1-2*, both of which contain two conserved CHORD domains typical of RAR1 proteins. The proteins differ by only four amino acids within these domains, reflecting a high degree of structural conservation. Phylogenetic analysis indicates that HbRAR1s are most closely related to MeRAR1, confirming the evolutionary conservation of RAR1 across plant species (Muskett et al., 2002; Bieri et al., 2004). These findings support the hypothesis that RAR1 proteins play a conserved role in plant immunity.

In plants, HSP90, RAR1, and SGT1 form a dynamic chaperone complex that stabilizes NLR receptors and maintains their signaling activity (Seo et al., 2008; Ito et al., 2015). In *Arabidopsis* and barley, RAR1 binds the HATPase domain *via* its CHORD-I domain, whereas SGT1 associates with the same domain through its CS region, and both CHORD domains also interact with SGT1 (Takahashi et al., 2003). By contrast, our results in rubber tree indicate that both CHORD domains of HbRAR1 can interact with HbHSP90.1 and HbSGT1b, suggesting an adaptable interaction pattern that may have evolved to accommodate specific NLR clients. Biologically, this interaction diversity implies that the HSP90**–**SGT1**–**RAR1 chaperone machinery is fine-tuned for different immune receptors, rather than following a rigid one-size-fits-all mechanism.

In *H. brasiliensis*, HbHSP90.1 was previously shown to interact with HbSGT1b (Liang et al., 2023; Wang et al., 2023), but whether RAR1 is involved in this regulatory network was unknown. Here, Y2H and BiFC assays demonstrated that HbRAR1–1 and HbRAR1–2 interact with HbHSP90.1 and HbSGT1b. Domain mapping revealed that both CHORD-I and CHORD-II domains of HbRAR1 bind the HATPase domain and the CS domain. Interestingly, full-length HbRAR1 and HbSGT1b interact in both the cytoplasm and nucleus, while HbRAR1**–**HbHSP90.1 interactions predominantly occur in the nucleus. The HbSGT1b**–**HbHSP90.1 interaction displayed dynamic subcellular relocalization over time, and all domain-level interactions were confined to the nucleus. In tobacco, phosphorylation-mimicking mutations in the MAPK-targeted region of SGT1 promote its nuclear accumulation and restrict the N protein to the nucleus, thereby altering NLR nucleo-cytoplasmic partitioning (Hoser et al., 2013). The HSP90**–**RAR1**–**SGT1 complex has been implicated in both stabilizing and degrading NLRs to balance immune activation and repression (Azevedo et al., 2002; Kadota and Shirasu, 2012; Chapman et al., 2022). Thus, we propose that post-translational modification of HbSGT1b may regulate HbSGT1b**–**HbHSP90.1 localization and, consequently, modulate NLR-mediated immunity.

The interaction between HbRAR1 and HbHSP90.1/HbSGT1b is critical not only for maintaining NLR protein stability and conformational readiness but also for facilitating their activation and downstream defense responses.

Our previous research identified HbHSP90.1 and HbSGT1b as chaperones associated with powdery mildew resistance in the rubber tree. Specifically, HbHSP90.1 interacts with HbCNL2, a member of the same family as HbCNL4, playing a critical role in the accumulation and stability of HbCNL2 (Liu et al., 2022; Liang et al., 2023; Wang et al., 2023). This observation suggests a potentially similar mechanism for HbCNL4. Furthermore, literature reviews indicate that the HSP90**–**SGT1**–**RAR1 ternary complex is vital in other NLR protein-mediated hypersensitive responses. For instance, Zhang et al. demonstrated that the hypersensitive response mediated by the tomato Rx4 protein against *Xanthomonas euvesicatoria* pv. perforans race T3 relies on SGT1 and RAR1 (Zhang et al., 2020). In *N. benthamiana*, NbNRC2, NbNRC3, and NbNRC4 are NLR proteins integral to plant immunity, and research has shown that their proper function necessitates SGT1 and HSP90 (Dong et al., 2022). In this study, we demonstrated that HbCNL4 interacts with both HbHSP90.1 and HbSGT1b. Moreover, HR and ion leakage assays showed that the combined presence of HbHSP90.1, HbSGT1b, and HbRAR1 significantly amplifies HbCNL4-triggered cell death, indicating the ternary complex of HbHSP90.1**–**HbSGT1b**–**HbRAR1 plays a crucial role in the HbCNL4-triggered HR response through its interaction with HbCNL4.

However, the role of RAR1 in the regulation of NLRs may not be universal across all plant species or immune pathways. Previous studies revealed that some NLR-mediated resistances require both SGT1 and RAR1, others depend on only one, and some function independently of either protein (Oh et al., 2014; Chapman et al., 2021; Wu et al., 2025). For instance, barley MLA6-mediated resistance to powdery mildew requires both RAR1 and SGT1, whereas MLA1, despite 95% sequence identity with MLA6, requires neither (Azevedo et al., 2002). Tomato Sw-5b confers resistance to TSWV in an SGT1-dependent but RAR1-independent manner (Chen et al., 2021). This specificity in chaperone dependence underscores a nuanced layer of immune regulation in plants, whereby certain resistance pathways rely on general chaperone support while others have become self-sufficient. Importantly, such insights carry practical implications for crop improvement. In the context of rubber tree, knowing whether a candidate NLR gene requires the HSP90**–**SGT1**–**RAR1 complex can inform breeding strategies: by stacking or selecting immune receptors with differing co-chaperone requirements, breeders could ensure that no single pathogen strategy (e.g. targeting a co-chaperone) can extinguish all defenses, ultimately leading to more robust and durable disease resistance in *H. brasiliensis*.

Based on these findings, we propose a molecular model (**Figure 4E**). In rubber tree, HbRAR1 interacts with HbHSP90.1 and HbSGT1b through their two CHORD domains, binding to the HATPase domain of HbHSP90.1 and the CS domain of HbSGT1b, respectively. These interactions facilitate the assembly of the HbHSP90.1**–**HbSGT1b**–**HbRAR1 ternary complex. Furthermore, the core components of this complex, HbHSP90.1 and HbSGT1b, associate with HbCNL4, thereby maintaining the stability of the HbCNL4 protein and potentially playing a crucial role in HbCNL4-mediated HR. Our work provides new insights into the molecular mechanisms of disease resistance in rubber trees and offers a foundation for future efforts to exploit the HSP90**–**SGT1**–**RAR1 complex in improving crop resilience.

## Data Availability

The datasets presented in this study can be found in online repositories. The names of the repository/repositories and accession number(s) can be found in the article/**Supplementary Material**.
