# Correction: Functional characterization of HbRAR1 in *Hevea brasiliensis* reveals its role in the HSP90–SGT1–RAR1 complex during hypersensitive response

**DOI:** 10.3389/fpls.2026.1829105

**Published:** 2026-03-25

**Authors:** Qifeng Liu, Jiali Wang, Fei Yu, Yiying Lu, Yu Zhang, Meng Wang, Xiaoyu Liang

**Affiliations:** 1 State Key Laboratory of Tropical Crop Breeding, Sanya Institute of Breeding and Multiplication, School of Tropical Agriculture and Forestry, Hainan University, Sanya, China; 2 Huizhou Customs, Huizhou, Guangdong, China

**Keywords:** HbCNL4, HbHSP90.1, HbRAR1, HbSGT1b, *Hevea brasiliensis*, HR

The funder “the Project of Sanya Yazhou Bay Science and Technology City, (SCKJ-JYRC-2023-22)” was erroneously omitted from the **Funding** statement.

The original version of this article has been updated.

