# Supplementary material for: Functional characterization of HbRAR1 in Hevea brasiliensis reveals its role in the HSP90–SGT1–RAR1 complex during hypersensitive response
Source: Front Plant Sci. 2026 Feb 3;17:1751305. doi: 10.3389/fpls.2026.1751305 (PMC12909547; doi:10.3389/fpls.2026.1751305)
Supplement: Supplementary file 1 [file DataSheet1.docx]

**Support Information**

Qifeng Liu ^1^，Jiali Wang ^1^, Fei Yu ^2^, Yiying Lu ^1^,Yu Zhang ^1^, Meng Wang ^1*^, Xiaoyu Liang ^1*^

^1^ State Key Laboratory of Tropical Crop Breeding, Sanya Institute of Breeding and Multiplication, School of Tropical Agriculture and Forestry, Hainan University, Sanya 572025, China

2 Huizhou Customs, Huizhou, Guangdong, 516006, China

***Corresponding authors** E-mail: wangmeng@hainanu.edu.cn; liang2017@hainanu.edu.cn

**Support Figure**


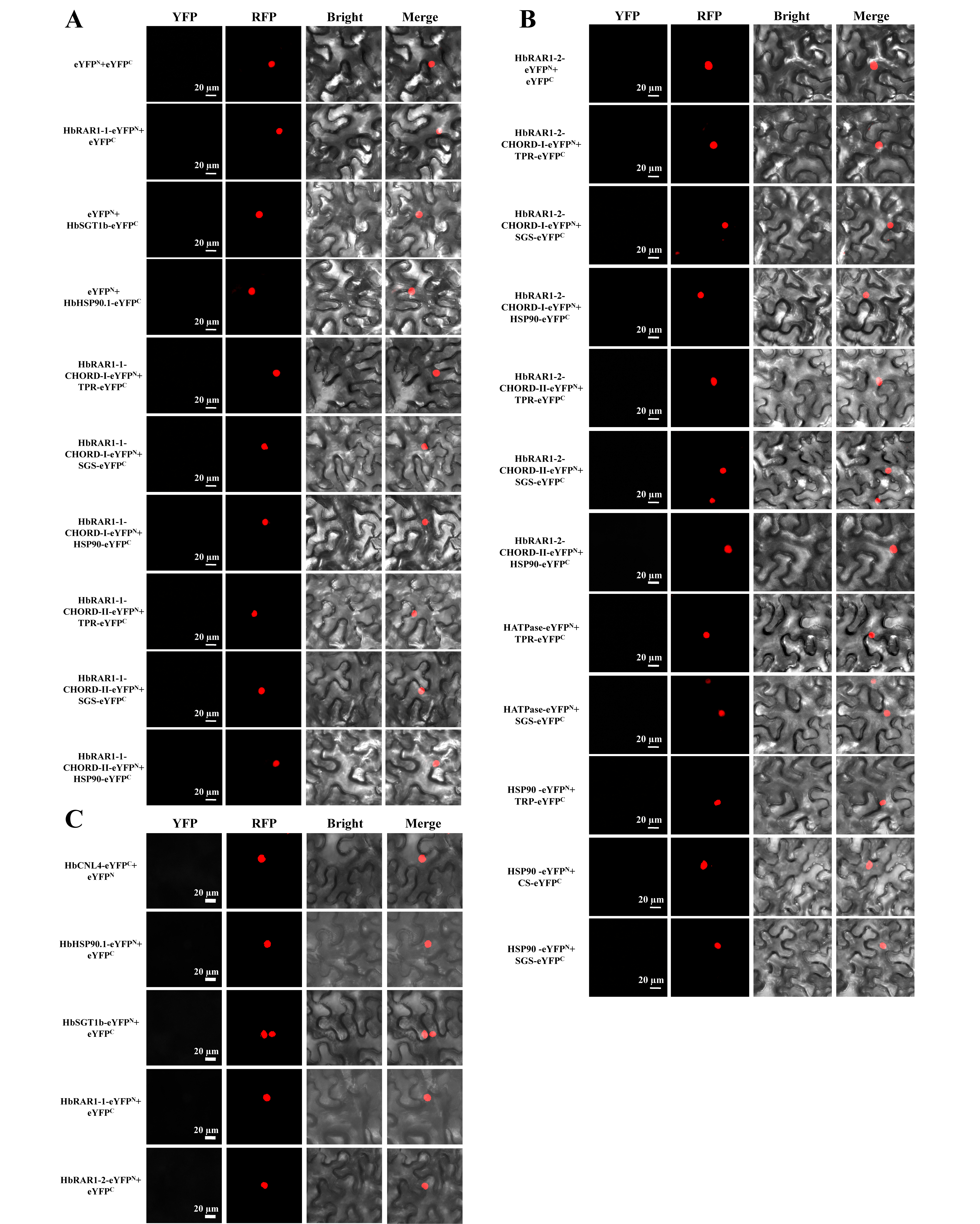


**Figure S1. Validation of interactions within the HbRAR1–HbHSP90.1–HbSGT1b complex and with HbCNL4.** (A) BiFC assay showing the interaction between HbRAR1-1 and HbHSP90.1 or HbSGT1b. (B) BiFC assay showing the interaction between HbRAR1-2 and HbHSP90.1 or HbSGT1b. (C) BiFC assay confirming the interaction of the HbRAR1–HbHSP90.1–HbSGT1b complex with HbCNL4. Scale bar, 20 μm.

**Table 1 Primers were used in this study**

| **primer name** | **Forward primer (5')** | **Reverse primer (3')** |
| --- | --- | --- |
| **HbRAR1-1-QP** | **ACTGCCTGTAGTTACCATCCC** | **CGTTGTGCCATCCCTTG** |
| **HbRAR1-2-QP** | **AGACATAAACCAGCCTCAAACC** | **CATCACAACATTTCCACCCTC** |
| **HbActin** | **GATGTGGATATCAGGAAGG** | **CATACTGCTTGGAGCAAGA** |
| **HbRAR1-1-GFP** | **TATGACCATGATTACGAATTCATGGAGCAGGAGGAGGTTGTT** | **CAGGTCGACTCTAGAGGATCCTTTTGTGACTAGATCCGCGTTG** |
| **HbRAR1-2-GFP** | **TATGACCATGATTACGAATTCATGGAGCGGGAGGTTGTTAGG** | **CAGGTCGACTCTAGAGGATCCAGACGCTGGATCAGCATTGTG** |
| **35S-GFP** | **CACAGGAAACAGCTATGAC** | **GTAAAACGACGGCCAGT** |
| **HbRAR1-1-eYFP^N^** | **CCCAGGCCTACTAGTGGATCCATGGAGCAGGAGGAGGTTGTT** | **CTCCTACCCGGGAGCGGTACCTCATTTTGTGACTAGATCCGCGTT** |
| **HbRAR1-2-eYFP^N^** | **CCCAGGCCTACTAGTGGATCCATGGAGCGGGAGGTTGTTAGG** | **CTCCTACCCGGGAGCGGTACCTCAAGACGCTGGATCAGCATT** |
| **HbHSP90.1-eYFP^N^** | **CCCAGGCCTACTAGTGGATCCATGGCGGATACAGAGACGTTTG** | **CTCCTACCCGGGAGCGGTACCTTAATCAACTTCCTCCATCTTGGA** |
| **HbSGT1b-eYFP^N^** | **CCCAGGCCTACTAGTGGATCCATGGCCAGCGAGTTGGCT** | **CTCCTACCCGGGAGCGGTACCTTATTCCCATTTGTTCATTACCATACC** |
| **HbSGT1b-eYFP^C^** | **TGGCGCGCCACTAGTGGATCCATGGCCAGCGAGTTGGCT** | **GTACATCCCGGGAGCGGTACCATATTCCCATTTGTTCATTACCATACC** |
| **HbCNL4-eYFP^C^** | **TGGCGCGCCACTAGTGGATCCATGGCAGATGGCGCTGTG** | **GTACATCCCGGGAGCGGTACCAAGGGTTTCAACCATTCGGG** |
| **35S-eYFP^N^** | **TGCCATCATTGCGATAAA** | **GATCGTTCAAACATTTGGC** |
| **35S-eYFP^C^** | **TGCCATCATTGCGATAAA** | **TCCCGGGATGTACCCATAC** |
| **HbRAR1-1^CHORD-I^-eYFP^N^** | **CCCAGGCCTACTAGTGGATCCATGGAGCAGGAGGAGGTTGTT** | **CTCCTACCCGGGAGCGGTACCGGGCTCGTTTATGTCAACTACCTT** |
| **HbRAR1-1^CHORD-II^-eYFP^N^** | **CCCAGGCCTACTAGTGGATCCCAGACCTGCAGGAATCGGG** | **CTCCTACCCGGGAGCGGTACCTCATTTTGTGACTAGATCCGCG** |
| **HbRAR1-2^CHORD-I^-eYFP^N^** | **CCCAGGCCTACTAGTGGATCCATGGAGCGGGAGGTTGTTAGG** | **CTCCTACCCGGGAGCGGTACCAGGCTGGTTTATGTCTACTACCATCTT** |
| **HbRAR1-2^CHORD-II^-eYFP^N^** | **CCCAGGCCTACTAGTGGATCCCAAACCTGCAGGAATCAGGG** | **CTCCTACCCGGGAGCGGTACCTCAAGACGCTGGATCAGCATT** |
| **HbHSP90.1^HATPase^-eYFP^N^** | **CCCAGGCCTACTAGTGGATCCATGGCGGATACAGAGACGTTTG** | **CTCCTACCCGGGAGCGGTACCAAGTTGGTCTTCCTTGAGGAATAGA** |
| **HbHSP90.1^HSP90^-eYFP^N^** | **CCCAGGCCTACTAGTGGATCCGAGTACCTTGAGGAGCGCCG** | **CTCCTACCCGGGAGCGGTACCTTAATCAACTTCCTCCATCTTGGA** |
| **HbSGT1b^TPR^-eYFP^C^** | **TGGCGCGCCACTAGTGGATCCATGGCCAGCGAGTTGGCT** | **GTACATCCCGGGAGCGGTACCATGTCTGTATTTTGGTTTTTCTGAAGA** |
| **HbSGT1b^CS^-eYFP^C^** | **TGGCGCGCCACTAGTGGATCCGAATACTACCAGAAGCCAGAGGAAG** | **GTACATCCCGGGAGCGGTACCTGCTCTTGATTTTGAAGATGGATATG** |
| **HbSGT1b^SGS^-eYFP^C^** | **TGGCGCGCCACTAGTGGATCCAAAGATTGGGATAAGTTGGAAGCC** | **GTACATCCCGGGAGCGGTACCATATTCCCATTTGTTCATTACCA** |
| **HbRAR1-1-pGBKT7** | **GCCATGGAGGCCGAATTCATGGAGCAGGAGGAGGTTGTT** | **CTGCAGGTCGACGGATCCTCATTTTGTGACTAGATCCGCG** |
| **HbRAR1-2-pGBKT7** | **ATGGCCATGGAGGCCGAATTCATGGAGCGGGAGGTTGTTAGG** | **CCGCTGCAGGTCGACGGATCCTCAAGACGCTGGATCAGCATT** |
| **HbSGT1b-pGADT7** | **GCCATGGAGGCCAGTGAATTCATGGCCAGCGAGTTGGCT** | **CAGCTCGAGCTCGATGGATCCTCAATATTCCCATTTGTTCATTACCA** |
| **HbHSP90.1-pGBKT7** | **ATGGCCATGGAGGCCGAATTCATGGCGGATACAGAGACGTTTG** | **CCGCTGCAGGTCGACGGATCCTTAATCAACTTCCTCCATCTTGGA** |
| **HbHSP90.1-pGADT7** | **GCCATGGAGGCCAGTGAATTCATGGCGGATACAGAGACGTTTG** | **CAGCTCGAGCTCGATGGATCCTTAATCAACTTCCTCCATCTTGGA** |
| **HbCNL4-pGADT7** | **GCCATGGAGGCCAGTGAATTCATGGCAGATGGCGCTGTG** | **CAGCTCGAGCTCGATGGATCCTCAAAGGGTTTCAACCATTCG** |
| **HbRAR1-1^CHORD-I^-pGBKT7** | **GCCATGGAGGCCGAATTCATGGAGCAGGAGGAGGTTGTT** | **CTGCAGGTCGACGGATCCGGGCTCGTTTATGTCAACTACCTT** |
| **HbRAR1-1^CHORD-II^-Pgbkt7** | **GCCATGGAGGCCGAATTCCAGACCTGCAGGAATCGGG** | **CTGCAGGTCGACGGATCCTCATTTTGTGACTAGATCCGCG** |
| **HbRAR1-2^CHORD-I^-pGBKT7** | **GCCATGGAGGCCGAATTCATGGAGCGGGAGGTTGTTAGG** | **CTGCAGGTCGACGGATCCAGGCTGGTTTATGTCTACTACCATC** |
| **HbRAR1-2^CHORD-II^-pGBKT7** | **GCCATGGAGGCCGAATTCCAAACCTGCAGGAATCAGGG** | **CTGCAGGTCGACGGATCCTCAAGACGCTGGATCAGCATT** |
| **HbHSP90.1^HATPase^-pGBKT7** | **ATGGCCATGGAGGCCGAATTCATGGCGGATACAGAGACGTTT** | **CCGCTGCAGGTCGACGGATCCAAGTTGGTCTTCCTTGAGGAATAGA** |
| **HbHSP90.1^HSP90^-pGBKT7** | **ATGGCCATGGAGGCCGAATTCGAGTACCTTGAGGAGCGCCG** | **CCGCTGCAGGTCGACGGATCCTTAATCAACTTCCTCCATCTTGGA** |
| **HbSGT1b^TPR^-pGADT7** | **GCCATGGAGGCCAGTGAATTCATGGCCAGCGAGTTGGCT** | **CAGCTCGAGCTCGATGGATCCATGTCTGTATTTTGGTTTTTCTGAAGA** |
| **HbRAR1-1-pGADT7** | **GCCATGGAGGCCAGTGAATTCATGGAGCAGGAGGAGGTTGTT** | **CAGCTCGAGCTCGATGGATCCTCATTTTGTGACTAGATCCGCG** |
| **HbRAR1-2-pGADT7** | **GCCATGGAGGCCAGTGAATTCATGGAGCGGGAGGTTGTTAGG** | **CAGCTCGAGCTCGATGGATCCTCAAGACGCTGGATCAGCATT** |
| **HbSGT1b^CS^-pGADT7** | **GCCATGGAGGCCAGTGAATTCGAATACTACCAGAAGCCAGAGGAAG** | **CAGCTCGAGCTCGATGGATCCTGCTCTTGATTTTGAAGATGGATATG** |
| **HbSGT1b^SGS^-pGADT7** | **GCCATGGAGGCCAGTGAATTCAAAGATTGGGATAAGTTGGAAGCC** | **CAGCTCGAGCTCGATGGATCCTCAATATTCCCATTTGTTCATTACCA** |
| **pGBKT7** | **TAATACGACTCACTATAGGG** | **ACTCTTAGGTTTTAAAACGAAAA** |
| **pGADT7** | **TAATACGACTCACTATAGGG** | **TGTGCATCGTGCACCATCT** |
| **HbCNL4-pBin** | **AATTCTGCAGTCGACCCCGGGATGGCAGATGGCGCTGTG** | **CAAGAAAGCTGGGTCCCCGGGTCAAAGGGTTTCAACCATTCG** |
| **HbRAR1-1-pBin** | **ATGGCCATGGAGGCCGAATTCTCACCCATAACTGGCCTGTTTC** | **CCGCTGCAGGTCGACGGATCCTTGGAGTAATTCAAGATGTACAAGATTTG** |
| **HbRAR1-2-pBin** | **GCCATGGAGGCCAGTGAATTCATGCCGGGTCTTGTCTCGG** | **CAGCTCGAGCTCGATGGATCCTCAACTTCTAAAACCCAGGCCA** |
| **HbSGT1b-pBin** | **GCCATGGAGGCCAGTGAATTCATGAAAAGGGAACACCCGAAT** | **CAGCTCGAGCTCGATGGATCCCTACTGCTTGTTGGCCAGCC** |
| **HbHSP90.1-pBin** | **GCCATGGAGGCCAGTGAATTCATGGCATCAAGCAGCAACCC** | **CAGCTCGAGCTCGATGGATCCTCATGCTATAAGGAACGGCAGA** |
| **pBin** | **GAATCTCAAGCAATCAAGCA** | **TACAAGAAAGCTGGGTCCCC** |
